# Inter-agency collaboration and disaster management: A case study of the 2005 earthquake disaster in Pakistan

**DOI:** 10.4102/jamba.v14i1.1088

**Published:** 2022-01-27

**Authors:** Ikram Shah, Tahir Mahmood, Sajjad A. Khan, Noor Elahi, Muhammad Shahnawaz, Adnan A. Dogar, Fazli Subhan, Khoula Begum

**Affiliations:** 1 Department of Development Studies, Faculty of Humanities, COMSATS University Islamabad, Abbotttabad Campus, Pakistan; 2 Department of Rural Development and Sociology, Karakorum International University, Daimir-Chilas Campus, Pakistan; 3 Department of International Relations, Abdul Wali Khan University Mardan, Khyber Pakhtunkhwa (KP), Pakistan; 4 Advanced Research Centre, Karakorum International University, Daimir-Chilas Campus, Pakistan; 5 Lasoona Project funded by Deutsche Welthungerhilfe, District Shangla, Khyber Pakhtunkhwa (KP), Pakistan; 6 Department of Agriculture and Food Sciences, Karakorum International University, Daimir-Chilas Campus, Pakistan

**Keywords:** disaster, disaster management, 2005 earthquake, inter-agency collaboration, ERRA, Pakistan

## Abstract

In post disastrous situations, coordinated and integrated interventions aimed at relief and rehabilitation not only help facilitate reaching out to the affected communities in a timely fashion but also pave the way to channel scarce and valued resources towards end users in an efficient and effective manner. This article attempts to trace the origins and gradual development of ‘inter-agency collaboration’ and the implications thereof for disaster management strategies in Pakistan through an analysis of relief and rehabilitation interventions undertaken by the Government of Pakistan in collaboration with local and international Non-governmental Organisations (NGOs) and relief agencies in the ex post of the 2005 earthquake. Data for this study were collected through structured and semi-structured interviews from government officials, representatives of NGOs and relief agencies and ordinary women and men in the earthquake stricken localities of Balakot and Mansehra districts of Pakistan. On the heels of the 2005 earthquake, both local NGOs and faith-based organisations in concert with international NGOs and relief agencies from around the world rushed to assist Pakistan in it’s rescue and relief operations at a time when the country was faced with the twin dilemma of both the non-existence of peculiar institutional arrangements for disaster management and a lack of the necessary technical and financial resources. The aftermath of the 2005 earthquake offered opportunity to the Government of Pakistan and the NGOs and relief agencies alike to transform their individual interventions into a robust and organised ‘inter-agency collaboration’, which was later on realised in the form of establishment of a national disaster management organisation called the ‘Earthquake Reconstruction and Rehabilitation Authority (ERRA)’. The establishment of ERRA not only paved the way for avoiding duplication and wastage of resources but also ensued in reaching out to the affected communities in a timely fashion. The Pakistani case offers implications in terms of highlighting the salience of establishing ‘inter-agency collaboration’ in other settings.

## Introduction

The early 21st century has witnessed a multitude of natural calamities ranging from massive earthquakes and cyclones through to disastrous tsunamis. The human and physical costs emanating from such disasters have been colossal (Guha-Sapir, Below & Hoyois 2015; OECD 2003; UN 2010). Asia, in particular, has remained the epicentre of natural calamities such as the Asian Tsunami (2004), Pakistan’s Earthquake (2005), Myanmar’s Cyclone Nargis (2008), China’s Wenchuan Earthquake (2008), Pakistan’s floods (2010) and Japan’s Tsunami (2011). The ravages of such calamities warrant the establishment and promotion of robust relief collaboration at regional and global levels alike (Lai et al. 2009; Yi & Yang 2014). During the past few decades or so, South Asia alone has witnessed around 1333 disasters, which wreaked havoc not only on physical infrastructure (causing devastation of assets worth US$105 billion) but also took the lives of more than 980 000 people. Ironically, these disasters also pushed around 2.4 billion people into a state of utter vulnerability (SAARC 2011). At such critical junctures, uncertainty and inefficient service delivery have served to exacerbate the predicament of those who had already paid a huge price. During the 21st century, host country governments, civil society organisations (CSOs), international non-governmental organizations (INGOs) engaged in disaster management have put an overarching emphasis on fostering planning and establishing collaboration amongst various jurisdictions and organisations in order to help substantiate the capabilities of disaster torn countries to respond swiftly and effectively to disastrous situations (Berman & Korosec 2005; Lai 2012; Nolte, Martin & Boenigk 2012; UNISDR 2005).

Collaboration is both desirable and necessary for coping with the ravages of natural disasters (Bryson, Crosby & Stone 2006) that can barely be managed single handedly by host countries, international organisations or non-governmental organization (NGOs) (Agranoff & McGuire 2004). Collaboration refers to the interaction between actors who work together in anticipation to attain mutual interests through accomplishment of a set of complex goals and a collective responsibility for interconnected tasks, which cannot be accomplished individually (McNamara 2012). Inter-agency collaboration, on the contrary, generally entails partnership amongst government, business, non-profits and philanthropists, communities and/or the public as a whole for achieving a common goal through pooling of various resources, shared decision-making and accountability (Bryson et al. 2006; Kamensky 2004). Collaboration amongst key players is an integral component of emergency response. The scope and nature of collaboration is determined by two important factors, that is, the needs of disaster-affected communities and the goals of collaborating actors (Kapucu & Garayev 2011). Furthermore, collaboration also entails establishing partnerships, either short term or long term, in multiple areas or across different levels of government to address a wide variety of social problems (Comfort, Boin & Demchak 2010; Kapucu 2012; Quick & Feldman 2014). During the past two decades or so, inter-agency collaboration has gained much attention amongst scholars, policymakers and professionals to grapple with the most challenging societal problems (Ansell & Gash 2008; Bryson et al. 2006; McGuire 2006; Rethemeyer 2005). Notwithstanding, the emergence of a voluminous literature on inter-agency collaboration our understanding of how communities recover from disastrous events through inter-agency collaboration is still naive (Comfort & Kapucu 2006; Emerson, Nabatchi & Balogh 2012; Kapucu 2006). This article attempts to contribute to the literature that emphasises the role of inter-agency collaboration in natural disasters through an analysis of the processes and formation of collaboration in the October 2005 post-earthquake Pakistan.

Amongst natural hazards, earthquake stands out as the most disastrous calamity, which wreaks havoc to physical infrastructure and subsequently turns the socio-economic fabrics for a society into topsy-turvy (Cooper, Donnelly & Johnson 2011; Dore & Etkin 2000; Hallegatte & Przyluski 2010). Before the onset of the October 2005 earthquake, disaster management in Pakistan, like other developing countries, had received little if any attention. Consequently, the 2005 catastrophic earthquake played out as a challenge for the government of Pakistan in terms of responding to and recovering from the ravages of the so-called earthquake. On the heels of the 2005 earthquake, the Government of Pakistan sought to secure both technical and financial assistance from international community. The call prompted a large number of organisations, both national and international, to come to the rescue of the disaster-affected communities. Notwithstanding, bestowed with massive financial assistance, the Government of Pakistan was faced with the dilemma of how to coordinate the activities of different actors in a disaster situation. This study employs the Bryson et al. (2006) Inter-agency collaboration model (ICM) as a framework of analysis to trace the origins, establishment and nature of collaboration between or amongst the Government of Pakistan and other actors. The ICM comprises five key components, that is, initial condition, process dimension, structure and governance, contingencies and constraints, and outcomes and accountability. Inter-agency collaboration model has been extensively applied to various forms of partnerships and collaborations between key players including host country governments, NGOs and local communities in disastrous situation (Lai 2012; Simo & Bies 2007).

This study conceptualises ICM as a relationship established between Earthquake Rehabilitation and Reconstruction Authority (ERRA, an organisation established by the Government of Pakistan in the ex post of the 2005 earthquake) and international non-governmental organisations (INGOs) during 2005 earthquake response and recovery phases. The article specifically emphasises probing the effectiveness of inter-agency collaboration in disastrous situations such as the 2005 earthquake. The analysis in this article demonstrates how inter-agency collaboration ensued in radically overhauling existing institutional arrangements associated with disaster management in Pakistan and facilitated the collaborators to effectively cope with ravages of the October 2005 notorious calamity.

### Inter-agency collaboration in disaster management

Over the last two decades or so, the scale and scope of natural and man-made disasters have dramatically increased. The ineffectiveness of traditional disaster management approaches has given way to the emergence of decentralised emergency management systems. This shift is being prompted by the exigency to collaborate during various stages of a disastrous situation (Kapucu & Garayev 2011). Inter-agency collaboration serves as an integral component of modern disaster management (Waugh & Streib 2006). Disasters, be that natural or man-made, open up window of opportunity for bringing multiple actors together. More often than not, such actors not only belong to different nationalities but also vary considerably in terms of their capacities, values, norms and objectives (Kettl 2008; Mitchell 2006). The effectiveness and efficiency of such organisations, however, largely depend upon the level of collaboration in disaster situations (Bryson et al. 2006; Drabek & McEntire 2002; Vangen & Huxham 2003). The responding agencies with limited capacity and capabilities seek to establish partnerships to pool their various resources, sharing of expertise and information for a larger collaboration in any disaster (McDonald 2008). Collaboration might take on a variety of forms such as transboundary collaborative response by voluntary organisations during Asian Tsunami 2004 (Lai 2012), intergovernmental collaboration during Wenchuan earthquake in China 2008 (Guo & Kapucu 2015) and Hurricanes Katrina and Rita in 2005 (Simo & Bies 2007) and multi-organisational collaboration in 2004 post-tsunami Tamil Nadu, India (Raju & Becker 2013). The success of different variants of collaboration, however, depends not only on the capacities of collaborative agencies but also on how adequately they collaborate during different phases of disasters (Samba 2010).

Large-scale calamities often pose considerable challenges to responding agencies such as non-profit organisations, the private sector, volunteer organisations and even community groups with varying capacities and capabilities (Kulatunga 2011). At times, these actors face difficulties in undertaking their roles and responsibilities particularly when the skills and capacities of other collaborating agencies are unbeknown to them. Such assorted resources may be a challenge for responding agencies (Gryszkiewicz & Chen 2010). Collaboration is generally regarded as a panacea to cope with the issue of poor performance (Eide et al. 2013). In order to avoid duplication of activities and to effectively channelise available resources, it is imperative to institutionalise the coordination process (Raju & Van Niekerk 2013).

### Disaster management in Pakistan

Until the 2005 earthquake, like other developing countries, disaster management in Pakistan had received little if any attention until at least the onset of the 2005 earthquake. Disaster management was conceived of as an area that remained in the shadows in mainstream development planning process. Consequently, disaster management departments or organisations in Pakistan largely remained under-resourced, incapacitated, lacked administrative experiences and hierarchal structure extending from national to local levels (Mustafa 2001; National Disaster Management Authority [NDMA] 2010). Before the onset of the 2005 earthquake both a coherent national policy framework and institutional arrangements of the sort required to cope with mega disasters were non-existent in Pakistan (Cheema, Mehmood & Imran 2016; NDMA 2010). Around 27 organisations (both Federal and Provincial) with overlapping roles and responsibilities were supposedly involved in disaster management (Cheema et al. 2016). Similarly, the entire disaster management system largely hinged on ‘Emergency Response Paradigm’ (ERP) (UN 2010).

Historically, disaster management policies in Pakistan were parochial in nature because such policies emphasised exclusively on a particular type of hazard such as flooding and hence entailed activities associated only with floods, that is, flood rescue and other relief operations (NDMA 2010). From 1947 to 2014, floods in Pakistan have played out as a recurrent phenomenon, which have ensued in cumulative losses of US$39 billion (from 25 major floods) (Guha-Sapir et al. 2015). The Pakistan Metrological Department founded in 1947 and the Flood Forecasting Division were the institutional arrangements for emergency situation in Pakistan. Subsequent flooding in East Pakistan resulted in the Climate Act 1958, which stipulated the roles and responsibilities of the state in disastrous situations. The act was mainly based on response and relief efforts to facilitate the affected communities with no mechanism for prevention and preparedness (Cheema et al. 2016; NDMA 2007). Similarly, Civil Defence established in 1953 was envisaged to deal with emergencies and to protect the general public during natural calamities. The Emergency Relief Cell (ERC) established at the federal level during 1971 embodies yet another institutional arrangement to serve as a helping hand in cyclone hit Eastern Pakistan. The ERC was responsible for post-disaster situations including coordination with provincial relief departments. The Space and Upper Atmosphere Research Commission (SUPARCO), a federal government organization, established primarily for undertaking research on space related issues was also responsible for providing support for disaster management in Pakistan. National Crises Management Cell constituted in 1999 under the Anti-Terrorist Act at federal and provincial levels dealing with emergency situation work under the Ministry of Interior (MoI). The Water and Power Development Authority (WAPDA) (established in 1958), the Indus River Commission (established in 1960), the Federal Flood Commission (FFC) (established in 1977) and the Dames and Barrages Safety Council (1987), etc. epitomise the institutional arrangements purported to undertake relief activities under the Ministry of Water and Power (MoWP) (Cheema et al. 2016; NDMA 2010).

In addition, at provincial levels, different departments with varying capacities had the mandate to deal with disaster at the response stage. These departments included, for instance, Irrigation, Provincial Crises Management Cell, Police, Health, Agriculture and Livestock, Communication and Works, Food (responsible for stockpile of food items and management of ration depots for requirement of food items in affected areas) and Emergency Service-Rescue 1122 was a pilot project initiated in 2004 in Lahore, the capital city of Punjab province and later on extended to other districts. The model is also replicated by other provinces in anticipation to cope with emergency situations (Cheema et al. 2016).

### Pakistan’s earthquake in 2005

In October 2005, a moment magnitude (*M*_*w*_) 7.6 (US Geological Survey) earthquake struck the capital, northern Pakistan and the Kashmir region. The epicentre of earthquake was located 100 km north-northeast of Islamabad. This devastating earthquake was followed by 1000 aftershocks of (*M*_*w*_) of 5.0–6.0 in India-Pakistan Kashmir region. The Pakistan-administered Kashmir known as Azad Jammu and Kashmir (AJ&K) and the eastern districts of Khyber Pakhtunkhwa (KP) province were amongst the most severely affected regions (Asian Development Bank-World Bank [ADB-WB] 2005). The disaster left around 73 338 people dead, 69 412 severely injured and 3.5 million people homeless (ERRA 2006). The earthquake caused 70-km long surface rapture and 7-m vertical separation near Balakot, Bagh and Muzafarabad. The ravages that followed were thus unprecedented in the history of the Indian-Kohistan Seismic Zone (IKSZ) and Himalayas (Hussain & Yeats 2009; Kaneda et al. 2008; Yeats, Kausar & Nakata 2006). In Himalaya, an earthquake of this magnitude has long been anticipated (Bilham 2004). The preliminary damage assessment conducted by ADB and WB demonstrates that the earthquake had wreaked havoc on an area of 30 000 sq. km area comprising 9 districts, 35 tehsils and 400 villages was overall affected. Massive damages to government buildings and communication infrastructure were reported in earthquake hit areas. For instance, 796 health facilities, 6298 schools and colleges, 600 000 houses were destroyed and 2393 km roads were damaged (ADB-WB 2005). In terms of the scale of devastation, the 2005 earthquake overshadowed even the most notorious disasters in Pakistan such as the windstorm of 1965, which had claimed around 10 000 lives (World Bank 2014). The WB and ADB estimated $5 billion for the reconstruction and rehabilitation of damaged infrastructure in affected areas (ADB-WB 2005; Durrani at el. 2005).

## Methods

Hazara Division, the epicentre of the 2005 earthquake and probably the most severely affected region, constitutes the study areas for this research. The Bryson et al. (2006) model was employed to get an in-depth understanding of how inter-agency collaboration between government departments particularly ERRA and INGOs was established in post-earthquake 2005. Respondents belonging to both ERRA and INGOs (such as World Vision, International Rescue Committee, Islamic Relief and United Nations Office for Coordination on Humanitarian Assistance) were selected through snowball sampling technique (non-probability). This method helped us in the identification of relevant respondents, who were in one way or the other directly involved in inter-agency collaboration established in the ex post of the 2005 earthquake. All respondents were involved in the long-term rehabilitation and reconstruction process either at district, provincial or national level.

Twenty two semi-structured interviews were conducted between January and March 2017. Four semi-structured interviews were conducted with World Vision representatives, five respondents from the International Rescue Committee (IRC), five respondents from Islamic Relief and eight officials were interviewed from ERRA. All interviews were conducted in person and each interview took one and a half hour; a prior permission was taken from respondents for recording and documentation. More than two rounds of semi-structured interviews were conducted with five respondents from ERAA, IRC and United Nations Office for the Coordination of Humanitarian Affairs (UNOCHA), two rounds of semi-structured interviews were conducted with three respondents from ERRA, Islamic Relief and World Vision whilst the rest of semi-structured interviews were conducted in one round. An interview guide was used when conducting semi-structured interviews. The open question of the interview guide helped to keep interviews within the scope of the study by directing the respondents towards talking about the inter-agency collaboration. The interview allowed the respondents to speak freely within the specific themes whilst explaining the inter-agency collaboration. During the data collection, the interviewer asked probing questions to clarify ideas and explore more in-depth information. The important aspects covered in interviews included, for instance, (1) initial condition (2) process dimension (3) structure and governance (4) contingencies and constraints and (5) outcomes and accountability. Secondary data were retrieved from published reports, government briefings and feedback from UN and donor agencies. Before the earthquake in 2005, FFC, Pakistan Agriculture Research Council (PARC) and many international organisations such as ActionAid, Catholic Relief Service, Concern Worldwide, Oxfam, United Nations Food and Agriculture Organization (UNFAO), United Nations World Food Program (UNWFP), World Health Organization (WHO), United Nations Children’s Fund (UNICEF), United Nations Development Fund (UNDP) and Department for International Development (DFID) were engaged in the planning, implementation and oversight of programmes and projects for disaster risk mitigation (NDMA 2007).

All the interviews were transcribed, and the data were analysed in specified themes under the research model. This method helped and contributed towards the understanding of establishing inter-agency collaboration in post-earthquake 2005 disaster management in affected areas of Pakistan.

## Results and analyses

The 2005 earthquake stands out as one of the worst disasters Pakistan has ever witnessed. A large number of international relief agencies swiftly responded to grapple with the ravages of this catastrophic event (ERRA 2006). Given the large number of relief agencies, establishing collaboration amongst such agencies turned out to be a hard nut to crack for both political and civil administration alike. This study employed Bryson et al. (2006) model as a framework of analysis to disentangle the inter-agency collaboration established in the post-earthquake 2005 situation between the Government of Pakistan’s flagship organisation ERRA and INGOs. The model comprises five major categories: initial conditions, process, structures and governance, contingencies and constraints, outcomes and accountability. The analysis in this article attempts to spell out each of these categories in the ex post of the 2005 earthquake.

### Initial conditions

In Bryson et al. (2006) model, the initial conditions are categorised into three components: (1) the general environment characterised by commotion in the wake of the disaster, (2) sector failure that signify single-sector inadequacies to cope with any disaster and (3) the antecedents which refer to the linking mechanism and general agreement on the problem. Given the scale of havoc and commotion it inflicted in the region, the 2005 Earthquake is generally believed to have overshadowed all the previous disasters in the country. The lack of preparedness and the tenuous institutional capacity further exacerbated the commotion. The mountainous terrain of Hazara Division turned out to be yet another factor impeding both the Government and other relief agencies to reach the scattered population. This warranted the pursual of a joint venture by the government and other relief agencies. In Mansehra the capital city of Hazara Division, a project coordinator from ERRA, whilst recalling the situation stated that:

‘[*A*]t that morning when this catastrophic event (earthquake) unfolded, everyone was taken aback. On the heels of the earthquake, provision of all sorts of public services and goods halted altogether.’ (Participant 1, approximately 46 years old, male, coordinator)

One of the representatives from INGOs in Balakot (the epicentre of the 2005 Earthquake) spoke about the initial conditions and highlighted that situation has worsened in the area. Just after the disaster the information received by local sources reported on large-scale human losses and damages to valuable assets and infrastructure. Respondents in the study area revealed that both the federal and provincial governments lacked the demonstrable capacity and vigour to properly respond to emergency situation, notwithstanding government officials, at large, were determined to identify and resolve the issues that ensued from the chaotic situation. The initial response of the government entailed the deployment of two Army Divisions for undertaking the rescue and relief operations in the disaster-stricken areas. This initial response pursued by government also brought into spotlight the incapacity and inefficiency of the relevant government departments. For instance, in Mansehra district alone, the relevant government departments grappled to respond to the situation.

The 2005 earthquake had received considerable attention and coverage from both national media and international news agencies around the world, which brought into spotlight both the government’s institutional incapacity and inefficiency and the need for undertaking measures aimed at establishing inter-agency collaboration:

‘In compulsion the government had initiated the inter-agency collaboration for initial response … because, government alone could have barely responded to the situation. Furthermore, people in the earth stricken areas were also looking forward to seek assistance from relief agencies.’ (ERRA, Official)

Discursively, a variety of factors account for engendering collaboration between government and aid providing actors such as coping with rescue and rehabilitation issues in the ex post of the disaster, efficient and effective exploitation of available resources, avoiding duplication and overlapping of activities and taking advantage of the prowess and expertise of international aid and relief agencies. Majority of the respondents, 18 of them, highlighted that major international organisations arrived with its full capacity but to step in on foreign land was a major challenge in offering relief assistance. For instance, the IRC, World Vision and Islamic Relief had both experienced and relief specialists who could facilitate people in affected areas. An interview conducted with the IRC representative in health project indicated that the IRC had organisational capacity, skilled manpower and good resources to respond to disastrous situation of 2005 earthquake. In Mansehra, the field coordinator of World Vision recalled that the organisation was capable of establishing liaison amongst key stakeholders and bringing them on one platform. Non-profit organisations were closely affiliated with other central actors such as, UN agencies, large faith-based entities, universities and public sector organisations towards collective involvement in relief efforts.

Two respondents from ERRA described that although establishment of inter-agency collaboration was not free from challenges but the need for collaboration with capable players particularly with INGOs was felt within the government and it was realised that without involving these actors, government on its own could not reach to affected areas with full capacity. A dire need was felt between government and INGOs to establish regional level offices for coordination of the initial relief and rehabilitation interventions. In this way, the initial processes provided foundation for the formation of collaboration and core groups based on their experiences.

At the outset, international organisations such as the IRC, Islamic Relief, World Vision, etc. carried out their rescue and relief activities independently at large and demonstrated relatively more vigour and robustness in serving the affected communities upon receiving the No Objection Certificate (NOC) from the Government of Pakistan. The rescue and relief teams (majority of them were national level CSOs and faith-based organisations) arrived at Balakot city just after disaster delivered the essential life support goods and services to the earthquake survivors. Moreover, relief operations such as setting up of field hospitals, rescue operations, temporary shelters and distribution of food items were made possible because of relaxed government policy. Majority of organisations had formal agreements Memorandum of Understanding (MoUs) with the government of Pakistan. Therefore, the IRC, World Vision and Islamic Relief continued to extend relief efforts in Abbottabad, Mansehra and Batagram in Hazara Division. The IRC team leader recalled:

‘Operational manager called me after four days of earthquake and urged that collaborative working group will be needed to respond to this disastrous event.’ (IRC, Team leader)

### Process

Bryson et al. (2006) discussed the process component as the formal and informal agreement, leadership, legitimacy, building trust, managing conflict and planning. Here the focus is mainly on formal and informal structure development, legitimacy, trust and planning for relief and rehabilitation. According to ERRA officials, within the UN system, UNDP played catalytic role in providing financial resources, technical and adequate administrative support to United Nations Disaster Assessment and Coordination (UNDAC) and United Nations Office for the Coordination of Humanitarian Affairs (UNOCHA) for setting up the collaborative system in close partnership with government. For the first time the cluster approach was adopted including both UN and non-UN humanitarian organisations for responding to the emergency situation. Initially, nine core groups referred as clusters were established that aimed at strengthening the coordinative efforts. Although some administrative issues emerged from novelty of cluster system such as unclear guidelines, inter-cluster and intra-cluster coordination, leadership issues and inadequate representation of INGOs the cluster mechanism played an instrumental role in streamlining of resources, information sharing and reaching to earthquake survivors. Initially, on 10 October 2005 the government established the Federal Relief Commission (FRC) based at Prime Minister’s office, which was mainly responsible for rescue and relief collaborative efforts. The FRC had the responsibility for initial emergency response and channelising of resources. On 24 October 2005, ERRA was constituted with the mandate to supervise the coordinative efforts, residual relief operations and the reconstruction process. On 10 March 2006, the relief phase was formally pronounced to be over and the FRC was subsumed into ERRA.

Component of trustworthiness and legitimacy is the critical part of any collaboration. In the case of 2005 earthquake, the international organisations and IRC had working relationships with government in many development projects in different parts of the country. Therefore, these organisations had a good social image and track record, which put the government at ease to start collaborative initiative with partnering organisations. Managing conflict is a critical determinant of any inter-agency collaboration efforts, especially when majority of the partnering organisations belonged from diverse backgrounds. A total of 19 respondents maintained that all the collaborating partners have common agenda: protecting the earthquake affected communities with respect and dignity, minimising their suffering and bringing them back to normal life. The IRC representative revealed that in newly established collaborations, conflict among organizations usually arises due to the discrepancies associated with the organizations’ commitment, mission, interests, values and resources. However, in inter-agency collaboration, managing conflicts across organisations starts from the fact that when each partnering organisation recognises the other organisation’s commitment and values. This is particularly true in the case of post-earthquake 2005 collaborative initiatives, as many of private, public and non-profit organisations were securing their interests and mission but with passage of time these organisations realise that public needs are above their organisational interests and mission. Since ERRA had played a dominant role in the collaboration process, ERRA in tandem with its collaborative partners, thus, paid special attention to re-visiting the organizational commitment and values that were generally considered as antithetical to the common agenda.

Bryson et al. (2006) argued that inter-agency collaboration is more effective and successful when it is combined with emergent and deliberate planning. In 2005 earthquake, most of the initial planning at local level was made in a chaotic situation. In disaster response, the emergency efforts mostly rely on the existed planning and network at local level. But in Pakistan the *ex ante* planning (*if any existed*) failed to respond to the situation. Consequently, the proposition for establishing inter-agency collaboration was welcomed particularly by INGOs:

‘Without inter-agency collaboration responding to earthquake could be a miracle. In such a turbulent situation resources were mobilized in hurly-burly way. Because, either unnecessary supplies were received, or some got more while others were waited even for life saving drugs and food for survival.’ (World Vision representative)

Majority of the respondents revealed that the process of inter-agency collaboration is usually affected by political constrains and administrative problems. Although reaching to a formal agreement for establishing inter-agency collaboration mostly takes longer time but in 2005 earthquake government showed openness to a greater extent and high level of cooperation. One of the key aspects of the inter-agency collaboration was the establishment of knowledge-based collaboration amongst actors. Government initiative provides foundation for the vast documentation of information on risk assessment, lesson learned from various sectors by different stakeholders and contingency planning for disaster management. Respondents from ERRA and many others from INGOs thought that 2005 earthquake was one of the first natural disasters of that scale, it opened a new possibility for establishing a state level resource centre for disaster management in the country.

### Structure and governance

The structure and governance component of the model referred to the informal and formal membership, structural configuration and governance structure. Nineteen of the respondents highlighted that in establishing collaborative efforts, governance and structure have substantial importance. Earthquake Rehabilitation and Reconstruction Authority was the main officiating body responsible for all decisions including usages of funds and coordination and monitoring of resources in post-earthquake scenario. The Earthquake Rehabilitation and Reconstruction Authority had devised a mechanism which pave the way for devolving maximum powers to the Provincial Earthquake Rehabilitation and Reconstruction Agency (PERRA), the State Earthquake Rehabilitation and Reconstruction Agency (SERRA) and District Reconstruction Units (DRUs). This forum acted as a secretariat for provincial and state steering committees. The chief headed each committee, a secretary with representation from line departments and planning wing of ERRA. The committee had the mandate to approve the work plans and authority to accept the projects of up to 250 million rupees. Earthquake Rehabilitation and Reconstruction Authority was also responsible for ensuring required coordination and facilitations for other key actors including INGOs. The cluster forum paved the way for effective collaborative mechanism. The UN system particularly the UNOCHA was in close collaboration with government for integrated emergency response. As the UNOCHA aimed at strengthening collaborative partnerships, predictability and accountability of humanitarian assistance and defining the roles and responsibilities of humanitarian organisations, therefore, a platform was provided to international organisations for information sharing, mobilisation of resources, which minimise the overlapping and duplication of resources. UNOCHA has online centralised system for information sharing; all the implementing partners were responsible for uploading their intervention details on system. This system provided a multi-level information sharing platform to all relief assistance providing actors:

‘At the outset, despite the availability of ample resources, an organized and integrated relief assistance mechanism was yet to be established. This could cause overlapping and duplication of interventions activities undertaken by different agencies. Given the dire need to make all relief assistance efforts integrated and relatively more organized a cluster system was unveiled which served as a platform for all the implementing partners to share as well as to get access to the necessary information concerning relief assistance in the earthquake stricken areas.’ (IRC Representative)

Majority of the respondents from INGOs noticed that in the case of INGOs much of the governance mechanism relies on each organisation’s commitment and mandate in their respective areas. Moreover, the INGOs have the responsibility to regularly publicise their intervention details on website for instance, type of relief assistance provided, catchment area, number of beneficiaries covered and services in progress. However, some of the respondents from INGOs highlighted that majority of the information shared on websites on UNOCHA or organisation’s own website is no longer available either online or in physical form. The online information sharing system was an innovative method for monitoring and evaluation (M&E) and provided valid data to other partner organisations. Whilst the system paved the way for making relief information transparent and widespread amongst all contributing organisations, still it was characterised by major flaws. For instance, gap in information shared by implementing partners and ground work, follow up of interventions and lack of trust amongst partner organisations.

All the respondents note that every organisation has its own governance mechanism to operate its activities. In the case of 2005 earthquake, ERRA played an instrumental role in providing guidelines for defining the roles and responsibilities of aid providing actors. For instance, organisational values and commitments are valued by collaborating partners but ERRA guidelines were monitored and partnering organisations paid attention to these guidelines whilst operating in any disaster hit area. Majority of the respondents highlighted that in the inter-agency collaboration power was concentrated at the top, that is, ERRA and responsibilities were shared across collaborating partners. Each partner is anticipated to own the collaborative effort initiated by the government and implement its approved project within the time and share the progress of the operation on regular basis.

### Contingencies and constraints

Bryson et al. (2006) model suggested that for effective and successful inter-agency collaboration focus on service delivery partnerships, agreement on institutional logics and dealing with power imbalances amongst collaborative partners is required for collaboration. In extreme events such as 2005 earthquake, the contingencies and constraints affect the process, structure and governance. Majority of respondents from ERRA and INGOs observed that the post-earthquake scenario complicated the situation as the institutional logics resulted in a level of conflict and power imbalance amongst stakeholders. For instance, power imbalance was reflected between ERRA and lower established agencies such as PERRA, SERRA and DRUs. Moreover, a severe power imbalance was also observed between big non-profit players and small socially embedded organisations. In addition, because of disparity in resource endowment, small organisations have less representation of their voices therefore, in most cases power goes to those with more resources.

According to INGOs representatives, the IRC, World Vision and Islamic Relief all were running their projects from international donations received for earthquake. These organisations were the custodian of donations and public charity received on the basis of humanitarian assistance. Power resides with top management to decide a project according to the need identified. Each partner is anticipated to take ownership of their projects and regularly reported their progress. Whilst ERRA, as the main collaborative body, provided facilitation to the partnering organisations, other organisations with abundant funding resources dominated the situation. During emergency response and recovery phase, publicity of donor organisations was mandatory for implementing partners. In both relief assistance and reconstruction projects the acknowledgement of funding agency was displayed on monument plaque.

### Outcome and accountability

Outcome and accountability is the last category of the model. Outcome is discussed in three categories: public value; first, second and third order effects, whilst accountability pertains to input, process or outcomes. Bryson et al. (2006) stated that public value in inter-agency collaboration is more likely to be created and sustained when sectorial characteristics strengths are enhanced whilst also finding ways to attenuated weaknesses of each sector. This leads to first-, second- and third-order positive effects. The first-order effects refer to new learning and innovative strategies. Second-order effects occur when partnerships are extended in the form of new collaboration and joint efforts. Third-order effects is evident in the last stage of collaboration when results are discernible on the ground. Moreover, inter-agency collaboration may be more successful when there is a system of accountability such as cross-checking of gathered information and a valid system of data management.

All the respondents explained that the disastrous event of 2005 not only attracted public, private and non-profit organisations at regional and global levels but also received wide coverage from national and international media. Therefore, a huge influx of bilateral and multilateral donors and dozens of non-governmental and private organisations were arrived. This creates an opportunity for establishment of new partnerships, extended the collaborative efforts outside the formal boundaries, learning from experiences and designing strategy for effective inter-agency collaborative efforts. The collaborative efforts initiated in post disaster scenario provided mechanism for channelising and mainstreaming of relief resources such as, charitable donations was discernible immediately after earthquake as first-order effects. Majority of relief providing agencies including INGOs covered most of the deprived and ignored segment of affected community.

Our interviewees also explained that collaborative experiences had positive effects both at organisational and personal level. In essence, partner organizations steered such collaboration opportunities towards fostering capacity building such as improving communication skills and human resource development. The local staff found the inter-agency collaboration very productive and revealed that they had the opportunity to learn beyond their expectations from such collaboration. An ERRA official mentioned in an interview that such exposure was never been experienced in particularly how to expand the inter-agency partnerships in the time of extreme events when each organisation had come with its full capacity. The local organisations such as, community-based organisations (CBOs) also took advantages from this inter-agency collaboration. For instance, INGOs involved these organisations in implementation process, arranged workshops and trainings for their capacity building, which increase their capability to run small-scale projects. Consequently, big non-profit organisations created social capital for themselves. This form of new partnership emerged as second-order effects.

Fourteen of the respondents explained that the system of accountability is prerequisite for successful collaboration. However, in time of disaster, the new partnership often creates coordination problems and the aspect of accountability may be challenging for partnering agencies. These problems range from competition and tension amongst collaborative partners. In the case of 2005 earthquake, initially, the challenge for the inter-agency collaboration lies in overall management of collaborating partners during rescue and relief phases. For example, our interviewees explained that instead of focusing on relief and rescue operations most of the organisations were interested to show-off their relief work and gain recognition for their efforts.

Nine respondents three from ERRA and six from INGOs explained that novelty of inter-agency collaboration created few problems, which overall disturbed the accountability of collaborative efforts. Initially, because of lack of capacity and efficiency at government level, resources were not properly streamlined. Therefore, achieving accountability in relief work was a challenge for FRC. After the establishment of ERRA the principle of accountability was ensured in three ways. Firstly, policy regarding the provision of relief and reconstruction services were formulated and implemented. Secondly, relief resources were channelised and a selection criterion was established for funding applications. Thirdly, indicators based monitoring system and tracking of information through multilevel platform for the projects were employed. For instance, ERRA with the assistance of Department for International Development (DFID) had developed the M&E system for unified data system and for effective tracking of fund and its usages. The INGOs particularly, the World Vision, IRC and Islamic Relief had the capacity to implement and monitor their project on its own. Our interviewees explained that each organisation has its own M&E mechanism that ensures accountability of interventions undertaken. Moreover, the cluster system further strengthens the process by integrated approach and generated unified and valid data system that is to some extent ensure the accountability aspect of INGOs. Such complex mechanism of (M&E) and development of unified data system ensure the third-order effects of inter-agency collaboration.

## Discussion

The current research is based on Bryson et al. (2006) ICM by examining and explaining the collaborative efforts initiated by government and INGOs particularly focused on the role of ERRA and IRC, World Vision, Islamic Relief and UNOCHA. The study indicates that the 2005 earthquake was one of the most disastrous event unfolded in Pakistan during the early 21st Century. On the heels of this catastrophic event, the relevant institutions grappled to cope with the situation because of lack of the requisite capabilities. The magnitude of loses and damages were unprecedented but there was a common understanding amongst ERRA officials and INGOs representatives concerning the effectiveness of collaborative mechanism established during disaster. There was consensus amongst respondents that inter-agency collaboration was a key for effective disaster response as just after disaster number of relief providing actors were skyrocketed. Although, this huge influx of bilateral, multilateral donors, non-profit and public organizations were a magic bullet for 2005 earthquake disaster management but encountered several administrative and operational challenges.

The current research endeavour indicated that regardless of government swift response and openness in initial stages administrative and operational issues were emerged from disaster response management. The major issues highlighted in interviews were the streamlining of resources and establishment of collaborative mechanism with relief providing actors. As the political leadership and civil administration were not prepared for such disastrous event therefore the coordination mechanism confronted with major administrative and operational problems. Although, respondents have mentioned the government willingness and involvement remained marvellous at all levels in establishing inter-agency collaboration, but it is interesting to note that UN system and INGOs played substantial role in terms of providing technical and financial support to the government. Therefore, ERRA had clear guidelines for setting up goals and mandates for the inter-agency collaboration.

A lot of resources were invested in structuring effective inter-agency collaboration. Therefore, the cluster approach did increase the collaboration amongst relief providing agencies and limiting duplication of resources and played vital role as a facilitator within and amongst government and relief providing actors. Although field level coordination is a crucial part of collaboration efforts such as, efficient use of resources by expanding the service delivery to a greater number of affected communities. Therefore, adaptation of referral mechanism was amongst the driving forces which fierce the coordination process for instance, the IRC established traumatised centres and provided psycho-social support to number of patients referred by different organisations. It is also interesting to note that the novelty of cluster system confronted some major issue such as, less ownership of the coordination process at government level, lack of involvement of donors’ agencies, unclear guidelines and under representation of INGOs/NGOs. The study also indicated that other deriving forces also hamper the coordination process such as, competition over funding between actors dwindle the coordination process as most of actors wanting to work in the area and show-off their work.

Pakistan’s disaster response strategy was mainly based on reactive approach. The West Pakistan Calamity Act (1958) was a legal remedy whilst ERC (1971) was institutional support at federal level dealt with disasters. Other network of emergency response institutions with overlapping roles and responsibilities were also being operational in the country. This lack of institutional capacity and efficiency exhibited the need for appropriate policy and institutional arrangements for disaster management at federal and provincial levels. Therefore, National Disaster Management Ordinance (2006) was the first initiative for integrated disaster response mechanism which further paved the way for the establishment of NDMA, Provincial Disaster Management Authority (PDMA) and Federally Administered Tribal Areas Disaster Management Authority (FDMA) in the country. The 2005 earthquake, exposed the existing institutional arrangements and deficiencies at all levels of government to deal with mega disaster. The post-earthquake 2005 inter-agency collaboration played instrumental role in mainstreaming the disaster management in development planning in the country.

## Conclusion

The findings of the study demonstrate that inter-agency collaboration serves as an integral component of disaster management and may turn out to be a magic bullet particularly in situations when institutional capacities in host countries are tenuous. On the heels of any disastrous situation, timely provision of services always poses challenges not only to the government at all levels but also to the aid relief providing agencies as well. An effective way to cope with such challenges is to seek for collaboration between different levels of government and the national and international relief agencies. Inter-agency collaboration in disastrous situations requires participation and ownership from all actors. However, governments shall preferably play a leading role and take all the requisite steps in order to institutionalise such initiatives, which may help facilitate the responding agencies both in the short- and long-term disastrous situations. Although such inter-agency collaboration may be a platform for achieving a set of common goals, sharing of information, pooling of various resources and the lessons learnt but it could also serve as a very useful tool for spelling out both the governments’ and other collaborating actors’ roles and responsibilities.

The inter-agency coordination was established in a turbulent situation resulting from 2005 earthquake, the urgency and attention for the relief work was intensified whilst challenges remained and even increased with the passage of time. Therefore, inter-agency collaboration such as introduction of cluster system was an appropriate approach for creating connection within and amongst assistance providing actors. On initial stages some administrative and operational issues emerged from the inter-agency collaborative initiative as no framework existed in the country. Arguably, since its establishment, ERRA was the main pillar of inter-agency collaboration and was on the forefront of relief and recovery operations and it continues to play a vital role in the reconstruction and rehabilitation processes. The INGOs contributions in earthquake were paramount from rescue to reconstruction phases. As disaster management is a complex process that requires preparedness and capability at all levels, the 2005 earthquake thus not only exposed the ineffectiveness of different levels of government to deal with mega disasters but it also opened up windows of opportunities for institutionalisation of disaster risk management (DRM) and disaster risk reduction (DRR) strategies in the country. The post-earthquake 2005 inter-agency collaboration transformed the disaster management system in Pakistan in mainstreaming the disaster management in development planning.

The findings also shed light on the relevance of Bryson et al. (2006) framework to study the establishment and the nuts and bolts inter-agency collaboration in disastrous situations. However, this study provides only limited snapshot of vast and expanded inter-agency collaboration established during 2005 earthquake therefore, it may be interesting to compare the results with other inter-agency collaboration in similar settings. In addition, an interesting area for future research could be the challenges of inter-agency collaboration for a long-term reconstruction in disaster situation particularly during an earthquake.
